# Interleukin-33 contributes to both M1 and M2 chemokine marker expression in human macrophages

**DOI:** 10.1186/1471-2172-11-52

**Published:** 2010-10-19

**Authors:** Amrita D Joshi, Sameer R Oak, Adam J Hartigan, William G Finn, Steven L Kunkel, Karen E Duffy, Anuk Das, Cory M Hogaboam

**Affiliations:** 1 Department of Pathology, University of Michigan Medical School, Ann Arbor, MI, USA; 2 Centocor Inc., Radnor, PA, USA

## Abstract

**Background:**

Interleukin-33 is a member of the IL-1 cytokine family whose functions are mediated and modulated by the ST2 receptor. IL-33-ST2 expression and interactions have been explored in mouse macrophages but little is known about the effect of IL-33 on human macrophages. The expression of ST2 transcript and protein levels, and IL-33-mediated effects on M1 (i.e. classical activation) and M2 (i.e. alternative activation) chemokine marker expression in human bone marrow-derived macrophages were examined.

**Results:**

Human macrophages constitutively expressed the membrane-associated (i.e. ST2L) and the soluble (i.e. sST2) ST2 receptors. M2 (IL-4 + IL-13) skewing stimuli markedly increased the expression of ST2L, but neither polarizing cytokine treatment promoted the release of sST2 from these cells. When added to naïve macrophages alone, IL-33 directly enhanced the expression of CCL3. In combination with LPS, IL-33 blocked the expression of the M2 chemokine marker CCL18, but did not alter CCL3 expression in these naive cells. The addition of IL-33 to M1 macrophages markedly increased the expression of CCL18 above that detected in untreated M1 macrophages. Similarly, alternatively activated human macrophages treated with IL-33 exhibited enhanced expression of CCL18 and the M2 marker mannose receptor above that detected in M2 macrophages alone.

**Conclusions:**

Together, these data suggest that primary responses to IL-33 in bone marrow derived human macrophages favors M1 chemokine generation while its addition to polarized human macrophages promotes or amplifies M2 chemokine expression.

## Background

Macrophages play a pivotal role in regulating the initiation, amplification, and resolution of innate immune responses. Several diseases including atherosclerosis, diabetes, cancer, and rheumatoid arthritis are associated with a defect or alteration in macrophage function [1]. These cells arise in the bone marrow from granulocyte/macrophage progenitors, which are the precursors of monocytes. Monocytes exit the bone marrow and in turn respond to cytokines and chemokines during their recruitment into tissue where they differentiate into resident macrophages. Macrophages can be classically activated (i.e. M1) or alternatively activated (i.e. M2) based upon the response of these cells to the extracellular milieu [2]. In the presence of IFN-γ and PAMPs such as LPS, macrophages become M1 activated while in the presence of Th2-type cytokines such as IL-4 and IL-13, macrophages undergo alternative activation or are skewed toward an M2 phenotype [2]. Macrophage polarization dramatically alters the immune properties of these cells as evidenced by the potent anti-microbial properties of M1 macrophages versus the prominent tissue repair properties of M2 macrophages [2]. Although M1 and M2 markers have been more thoroughly characterized in mouse macrophages [3,4], human macrophages show selective soluble and cell membrane associated marker expression following exposure of these cells to M1 or M2 skewing conditions [2]. Examples of chemokines/chemokines receptor markers that are selectively upregulated under M1 conditions include CCR7 and CCL3 whereas CCL18 is selectively upregulated under M2 conditions in human macrophages [5]. Mannose receptor is a selective M2 marker in both mouse [6] and human [5] macrophages.

IL-33, similar to IL-1β and IL-18, is a member the IL-1 family that has a major role in innate immune responses driven through its receptor ST2 [7-10]. IL-33 is constitutively expressed by endothelial and epithelial cells and is found associated with chromatin in the nucleus [11]. IL-33 is released by these cells following necrotic cell death, hence this cytokine has been referred to as an 'alarmin' [12,13]. Several experimental models have shown that binding of recombinant IL-33 to ST2 leads to the induction of Th2 immune responses [14-16], but its effects on a number of non-adaptive immune cells have been well documented [17]. Specifically, a recent study addressed the effects of IL-33 on mouse macrophages and showed that this cytokine amplifies the expression of M2 markers in these cells [18]. Less is known about the immunomodulatory effects of IL-33 in human cells but this cytokine is known to enhance both Th1 and Th2 responses in human basophils, Th2 cells, iNKT cells, NK cells, mast cells, and eosinophils [19-22]. However, little is known about the effect of IL-33 on primary human macrophages.

Herein, we investigated whether human bone marrow-derived macrophages express ST2 and respond to IL-33, and whether the activation status of human macrophages alters the response by these cells to this cytokine. These studies showed that human macrophages express both the transmembrane form of ST2 (ST2L) and the soluble form of ST2 (sST2). Characterization of the effect of IL-33 on human macrophage activation suggested that it promoted M1 chemokine generation in naïve human macrophages but enhanced the expression of M2 chemokine markers in previously polarized macrophages, regardless of the prior polarization signals. Together, these data suggest that IL-33 has a direct effect on the expression of M1 and M2 chemokine markers in human macrophages.

## Methods

### Culture of human bone marrow-derived macrophages

An Institutional Review Board (IRB) at the University of Michigan Medical School approved the studies described below (Study Title: The effect of inflammatory disease states on growth, maturation, and gene expression patterns of bone marrow derived cells; Study eResearch ID: HUM00005609). Informed consent was not required for IRB approval of this study because bone marrow was collected at the University of Michigan Medical Center from patients undergoing various diagnostic tests and mononuclear cells and only the unused portions of these bone marrow samples were studied.

Each bone marrow sample was first subjected to a density gradient centrifugation over Ficoll-Paque™ Plus (GE Healthcare Bio-Sciences AB, Uppsala, Sweden) to isolate cells of myeloid origin. In order to establish a reproducible protocol for growing macrophages from human bone marrow, we tested several types of culture conditions, media, and growth factor combinations. The protocol that yielded both the greatest number and purity of macrophages is described herein. Briefly, bone marrow cells were plated onto 100 mm tissue culture dishes and 5 ml of Iscove's Modified Dulbecco's Medium (IMDM; Thermo Fisher Scientific Inc., Waltham, MA) supplemented with 10% fetal calf serum (FCS), 100 U/ml of penicillin, 100 μg/ml of streptomycin, β-mercaptoethanol, 25 ng/ml of macrophage colony stimulating factor (MCSF), 2.5 ng/ml of granulocyte monocyte colony stimulating factor (GMCSF), 50 ng/ml of stem cell factor (SCF), and 20 ng/ml of IL-3 was added. All cytokine growth factors were purchased from R&D Systems (Minneapolis, MN). Cells received 10 ml of fresh IMDM containing cytokine growth factors at day 3 after plating and cultured for a total of 7-10 days at 37°C in 10% CO_2_.

Prior to an experiment, spent media was removed from the cultured macrophages, the plate was washed with Ca^+2 ^and Mg^+2 ^free PBS to remove non-adherent cells, and the macrophage monolayer was incubated in Ca^+2 ^and Mg^+2 ^free PBS for 10 min on ice. Next, macrophages were dislodged via repetitive pipetting. The resultant cell suspension was centrifuged at 400-× g for 10 min. The cell pellet was suspended in IMDM containing FCS, 100 U/ml of penicillin, 100 μg/ml of streptomycin, and β-mercaptoethanol and plated at a density of 1 × 10^6^/ml in triplicate wells of a 6-well tissue culture plate.

### Flow cytometry of human macrophages

Bone marrow-derived human macrophages were washed once with buffer containing 0.2% BSA and 0.1% NaN3. FcR blocking reagent from Miltenyi Biotec was used to block Fc receptors. These cell suspensions were then stained with the one of the following antibodies or antibody combinations: FITC conjugated CD11b (BD Biosciences, San Jose, CA), PECy7-conjugated CD14 (eBiosciences; San Diego, CA), biotin-conjugated CD163 (BD Biosciences) followed by PECy5 conjugated streptavidin (BD Biosciences), or biotin-conjugated CD68 (BD Biosciences) followed by PECy5 conjugated streptavidin (BD Biosciences). Cells were fixed in 4% formalin and analyzed using Beckman Coulter FC500 and FlowJo Software (Tree Star Inc.; Ashland, OR).

### Effect of IL-33 on M1 and M2 chemokine expression by human macrophages

In the first series of experiments, macrophages were polarized toward either a M1 or M2 phenotype using distinct stimuli. To skew macrophages toward the generation of M1 chemokine markers, triplicate wells (in a 6-well tissue culture plate) containing 2.5 × 10^5 ^cells were stimulated with 100 ng/ml of IFN-γ (PeproTech; Rocky Hill, NJ) combined with 1 μg/ml of LPS (Sigma-Aldrich; St. Louis, MO) for 24 h. To skew macrophages toward the generation of M2 chemokines, triplicate wells containing 2.5 × 10^5 ^cells with the combination of 10 ng/ml of IL-4 and 10 ng/ml of IL-13 (both from R&D Systems) for 24 h. In other experiments, macrophages were incubated with 10 ng/ml IL-33 (IL-33^112-270^; R&D) alone for 24 h. To study the effect of IL-33 on previously polarized macrophages, macrophages were activated in M1 or M2 conditions for 24 h. These cells were washed and exposed to 10 ng/ml of IL-33 alone, IL-33 in combination with M1 or M2 macrophage skewing cytokines, or the M1 or M2 cytokines alone for an additional 24 h in culture. In all experiments, cell-free supernatants were removed and individual wells were processed for immunocytochemistry, protein analysis by Western blotting, or quantitative RNA analysis.

### Immunocytochemistry and Western blotting analysis of ST2

Macrophages were either fixed in 8-well Labtek tissue culture slides for immunocytochemistry or treated with protein lysis buffer in 6-well tissue culture plates prior to Western blotting analysis. Employing a standard immunocytochemistry technique, an anti-ST2 polyclonal antibody from ProSci (Poway, CA) was used to stain for ST2 in primary human macrophages exposed to media alone, IL-33, M1-polarizing mediators, or M2-polarizing mediators. IgG control groups were also included in this protocol. Standard Western blotting techniques were used to detect ST2L and sST2 in cytoplasmic and nuclear extracts from cultured naïve and M2-polarized macrophages.

### Quantitative RT-PCR

To analyze gene expression, RNA was extracted from adherent human macrophages using TriZol (Invitrogen Life Technologies; Carlsbad, CA). RNA was then converted to cDNA using Murine Moloney Leukemia Virus reverse transcriptase (Invitrogen Life Technologies). The resulting cDNA was analyzed by real time q-PCR using the ABIPRISM 7700 detection system (Applied Biosystems, Foster City, CA). Premixed primer/probe reagents for CCR7 and CCL3 were purchased from Applied Biosystems. The primers used to detect mannose receptor were ACC TCA CAA GTA TCC ACA CCA TC and CTT TCATCA CCA CAC AAT CCT C. The primers used for the detection of CCL18 were TAC CTC CTG GCA GAT TCC AC and TTA GAA GAG GTG GCC TCC AG. The following forward and reverse primers were used for the detection of ST2: (forward) CAG GAA AGA AAT CGT GTG T, (reverse) GCC AAG AAC TGA CTG CCT. To further distinguish the soluble form of ST2 (sST2) from the membrane associated form of this receptor (ST2L) the following primers were used. Soluble ST2: (forward) AGG CTT TTC TCT TGT TTC CAG TAA TCG G; (reverse) CAG TGA CAC AGA GGG AGT TCA TAA AGT TAG A. ST2L: (forward) AGG CTT TTC TCT TGT TTC CAG TAA TCG G; (reverse) GGC CTC AAT CCA GAA CAT TTT TAG GAT GAT AAC. A SYBR green PCR mix was used to amplify ST2, ST2L, sST2, mannose receptor, CCL18. The expression of GAPDH was used as internal control for each PCR reaction.

### Statistical Analysis

Analysis of variance (ANOVA) and the Dunnett's test were used to detect statistical differences between control and polarized human macrophages with and without IL-33 treatment. P values of ≤ 0.05 were considered statistically significant.

## Results

### M1 and M2 chemokine marker expression by human bone marrow-derived macrophages

**Figure 1:**
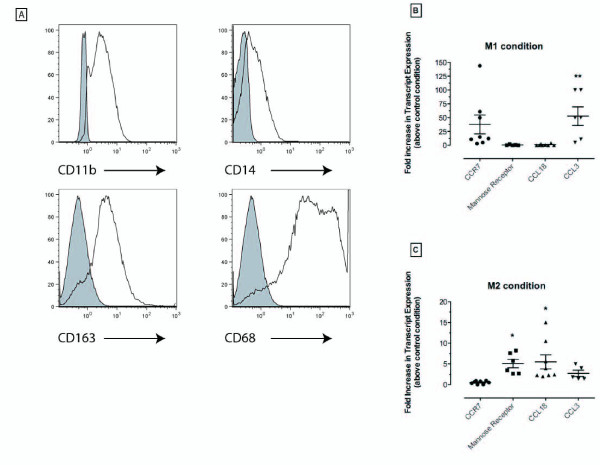
**M1 and M2 polarizing conditions in cultured human bone marrow-derived macrophages promote the expression of either M1- or M2-specific transcripts, respectively**. (**A**) Representative flow cytometric analysis of primary human macrophages derived from bone marrow. Cells were stained using anti-CD11b, anti-CD14, anti-CD163, or anti-CD68 antibodies. (**B**) Human macrophages stimulated with IFN-γ +LPS (i.e. M1 condition) or (**C**) IL-4+IL-13 (i.e. M2 condition) and gene expression was analyzed 24 h later by TAQMAN. Transcript levels for each M1 and M2 factor are expressed as fold increase over transcript levels of these factors in human macrophages exposed to media alone (i.e. control condition). Each symbol represents a single donor and macrophages from 5 to 8 bone marrow donors were analyzed; mean and SEM are also shown in panels **B **and **C**. * P ≤ 0.05 compared with transcript levels in control macrophages; ** P ≤ 0.01 compared with transcript levels in control macrophages.

Flow cytometric analysis of human bone marrow-derived macrophages revealed that the culture techniques employed promoted the expression of CD11b, CD68, and CD163 on approximately 90% of these cells (Figure 1A). CD14 was also expressed by approximately 40% of the cultured macrophages. Cultures of human macrophages were skewed toward a M1 phenotype upon exposure to IFN-γ and LPS for 24 h (Figure 1B). This was apparent by the elevated transcript levels of the M1 markers CCR7 and CCL3 in these cells (Figure 1B). The fold-increase in CCL3 transcript levels in M1 macrophages reached statistical significance compared with untreated or control macrophages (Figure 1B). Conversely, transcripts for two human M2 markers CCL18 and mannose receptor [5], but not the M1 markers CCR7 or CCL3 [5], were significantly elevated under M2 conditions compared with control macrophages (Figure 1C). Thus, with the appropriate external stimuli, human macrophages appeared to predominately express either M1 or M2 chemokine markers.

### ST2 expression by human macrophages exposed to either M1 or M2 conditions

**Figure 2:**
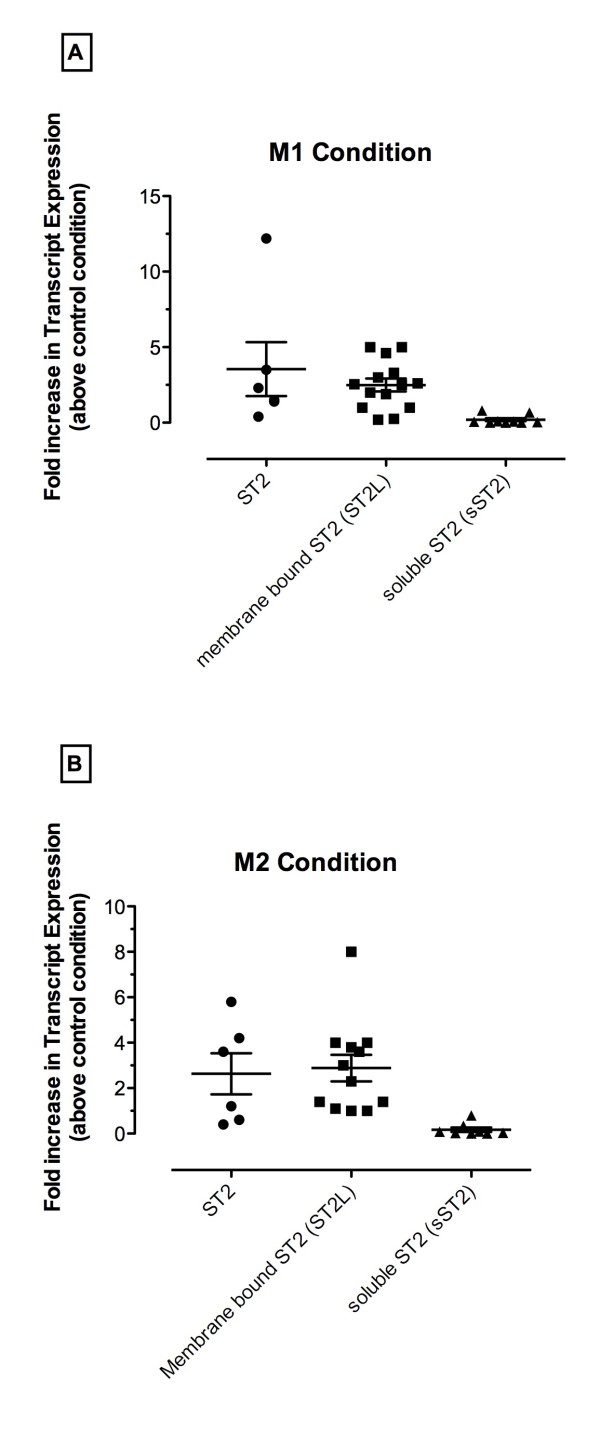
**ST2, membrane bound (ST2L), and soluble (s) ST2 transcript levels in M1 and M2 polarized human bone marrow-derived macrophages**. (**A**) Transcript levels of ST2, ST2L, and sST2 from macrophages exposed to M1 polarizing mediators. (**B**) Transcript levels of ST2, ST2L, and sST2 from macrophages exposed to M2 polarizing mediators. Results are expressed as fold increase in transcript levels compared with levels of these transcripts in human macrophages exposed to media alone (i.e. control condition). Each symbol denotes a single donor (n = 5-14 donors); mean and SEM are also shown.

Transcript and protein expression of ST2 was next determined in naïve, M1- and M2-polarized human macrophages. Quantitative PCR revealed that ST2 was constitutively expressed by macrophages but the exposure of these cells either to M1 (Figure 2A) or M2 (Figure 2B) conditions markedly increased the expression of ST2. Further quantitative PCR analysis revealed that the increase in ST2 expression was restricted to the membrane form of this receptor, namely ST2L and increases in soluble ST2 transcript levels were not apparent with either M1 or M2 conditions (Figure 2A &2B).

**Figure 3:**
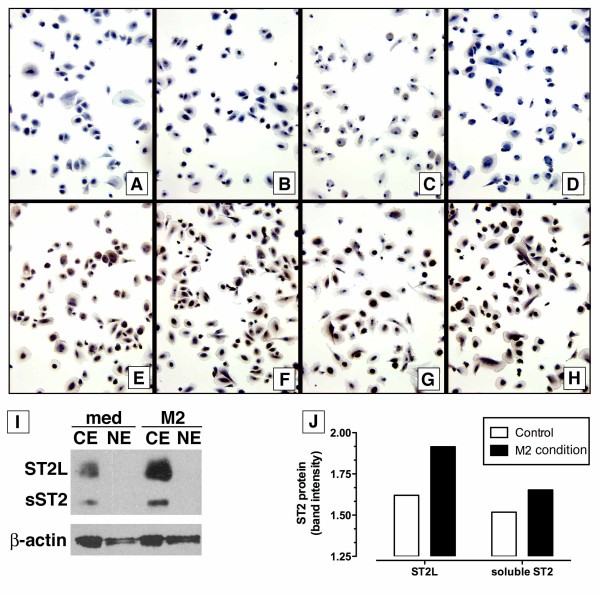
**ST2 protein expression in human bone marrow-derived macrophages**. (**A-H**) Immunostaining of human macrophages cultured on Lab-Tek slides after exposure to medium alone (A & E), IL-33 (B & F), M1 polarizing mediators (C & G), or M2 polarizing mediators (D & H). IgG controls are shown in panels A, B, C, & D. (**I**) Western blotting for ST2L and sST2 protein was performed in protein extracts from naïve and M2-polarized human macrophages; CE = cytoplasmic extract and NE = nuclear extract. (**J**) Quantitative analysis of ST2L and sST2 protein band intensity.

Immunocytochemical and Western blotting analysis of ST2L and sST2 protein expression in human macrophages are summarized in Figure 3. ST2L protein was present in naïve macrophages (Figure 3E), and in cultures of human macrophages exposed to IL-33 alone (Figure 3F), and when cultured in M1 conditions (Figure 3G) and M2 conditions (Figure 3H). Because the intensity of ST2L staining appeared to be greatest in M2-treated macrophages, Western blotting analysis was completed to compare expression in these macrophages with naïve or control macrophages. As shown in Figure 3I &3J, both ST2L and sST2 were increased in expression in M2 macrophages compared with the control (i.e. medium alone) group. Together these data showed that ST2 protein was present in human macrophages exposed to either M1 or M2 conditions, suggesting that both types of macrophages might be responsive to exogenous IL-33.

### Exogenous IL-33 blocked LPS-mediated induction of a M2 but not an M1 chemokine marker expression in human macrophages

**Figure 4:**
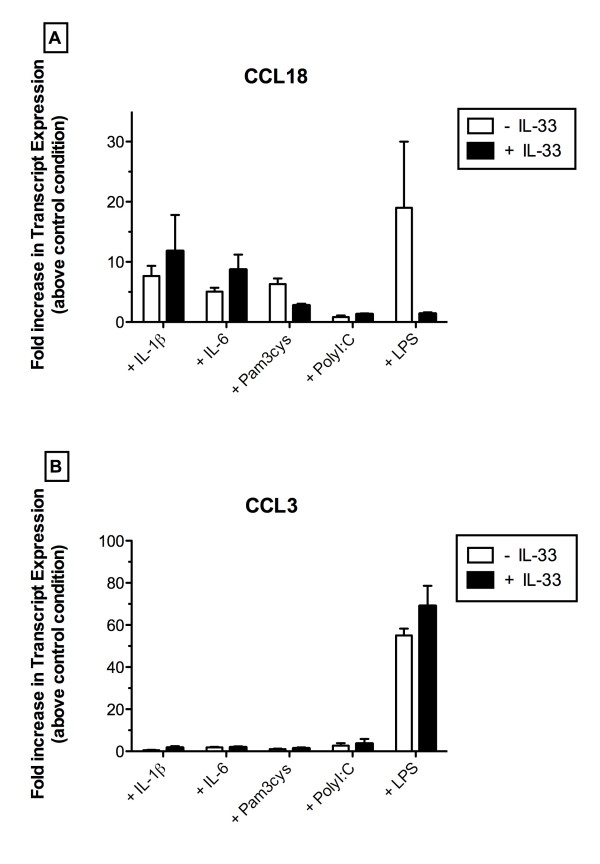
**Regulatory effects of IL-33 in LPS-activated human macrophages**. (**A**) CCL18 transcript levels in primary macrophages exposed to cytokine or TLR ligand alone or in combination with 10 ng/ml of IL-33. (**B**) CCL3 transcript levels in primary macrophages exposed to cytokine or TLR ligand alone or in combination with 10 ng/ml of IL-33. Results are expressed as fold increase in transcript levels compared with levels of these transcripts in human macrophages exposed to media alone (i.e. control condition). Data are expressed as mean ± SEM and are representative of experiments performed with 4 donor macrophage lines.

ST2 has been shown to sequester MyD88 (an adaptor molecule necessary for PAMP activation of cells) away from TLR4 thereby blunting LPS-induced activation of mouse macrophages [23]. To address whether ST2 activation similarly altered LPS responses in human macrophages, we next examined whether the addition of IL-33 to naïve human macrophages modulated LPS activation in these cells. CCL18 (Figure 4A) and CCL3 (Figure 4B) were selected as the prototypic M2 and M1 chemokine markers, respectively. IL-33 failed to markedly modulate IL-1β-, IL-6-, Pam3Cys-, or PolyI:C-induced expression of CCL18 but it completely blocked the LPS-induced expression of this chemokine (Figure 4A). Conversely, the addition of IL-33 to naïve macrophages did not block the ability of LPS to drive the transcript expression of CCL3 (Figure 4B). Thus, the inhibitory effect of IL-33 on LPS activation appeared to be restricted to a M2 but not a M1 chemokine marker in naïve human macrophages.

### Effect of IL-33 on macrophage M1 and M2 chemokine expression

**Figure 5:**
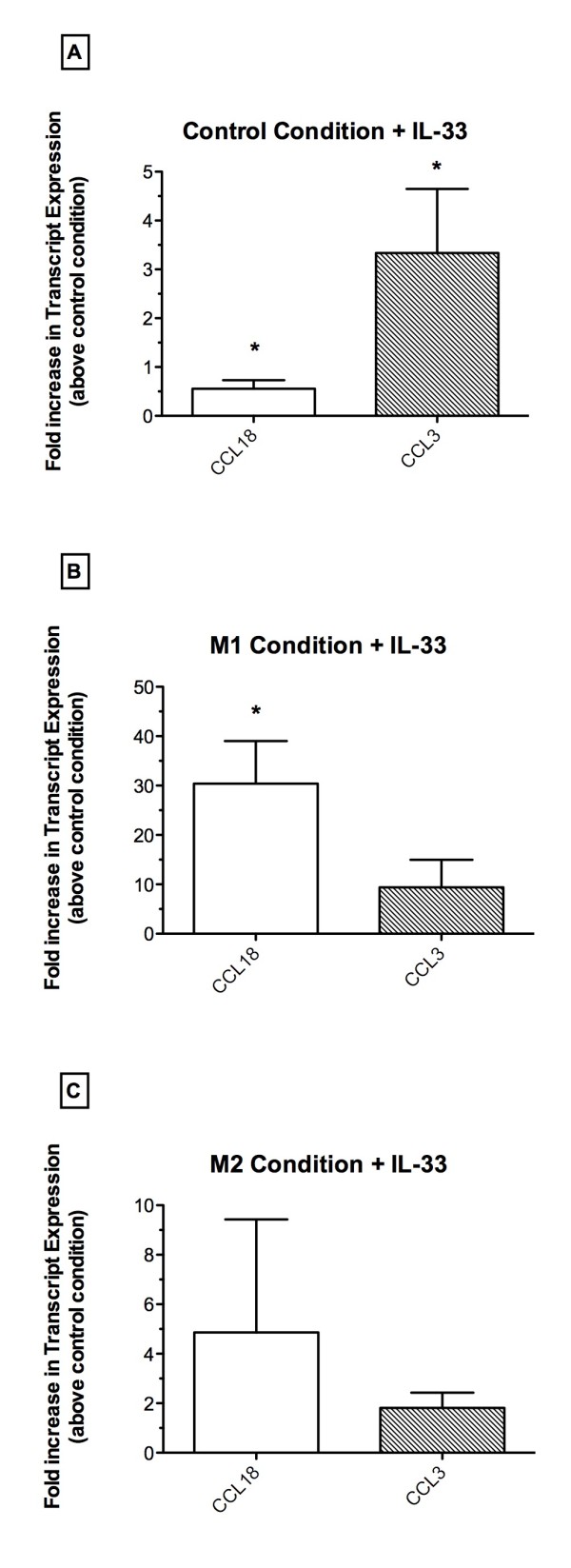
**Effect of IL-33 on human macrophage polarization**. (**A**) CCL18 and CCL3 transcript expression in macrophages following exposure to IL-33 (10 ng/ml) alone for 24 h. (**B**) CCL18 and CCL3 transcript expression in human macrophages following exposure to M1 polarizing mediators + IL-33 (10 ng/ml) for 24 h. (**C**) CCL18 and CCL3 transcript expression in human macrophages following exposure to M2 polarizing mediators + IL-33 (10 ng/ml) for 24 h. Results are expressed as fold increase in transcript levels compared with levels of these transcripts in human macrophages exposed to media alone (i.e. control condition). Data are shown as mean ± SEM and are representative of three separate experiments with 3 donor macrophage lines. * P ≤ 0.05 compared with transcript levels in appropriate control macrophages.

Because IL-33 had a differential effect on the expression of M1 and M2 chemokine markers in naïve macrophages, additional experiments were undertaken to explore this novel modulatory effects of IL-33 on human macrophages. As shown in Figure 5A, the addition of IL-33 alone to naïve macrophages significantly decreased the transcript expression of CCL18, while it significantly enhanced the transcript levels of CCL3 compared with transcript levels of these chemokines in untreated, naïve macrophages. When IL-33 was added with M1 polarizing mediators to macrophages, the transcript expression in these cells was dramatically shifted toward CCL18 expression; transcript levels of this chemokine were significantly increased approximately 30-fold above the M1 condition alone (i.e. control condition for this study) (Figure 5B). The addition of IL-33 to the M1 condition only modestly increased CCL3 transcript levels in these cultures. The addition of IL-33 with M2 polarizing mediators to human macrophages did not appear to skew these cells appreciably toward either CCL18 or CCL3 transcript expression since both chemokines were similarly increased above the M2 condition alone approximately 2-4 fold (Figure 5C). Thus, the addition of IL-33 to naïve macrophages favored the expression of an M1-associated chemokine marker over an M2-associated chemokine marker, but its addition to human macrophages cultured in M1 conditions promoted the expression of the M2 marker CCL18.

### Effect of IL-33 on human macrophages previously exposed to either M1 or M2 conditions

**Figure 6:**
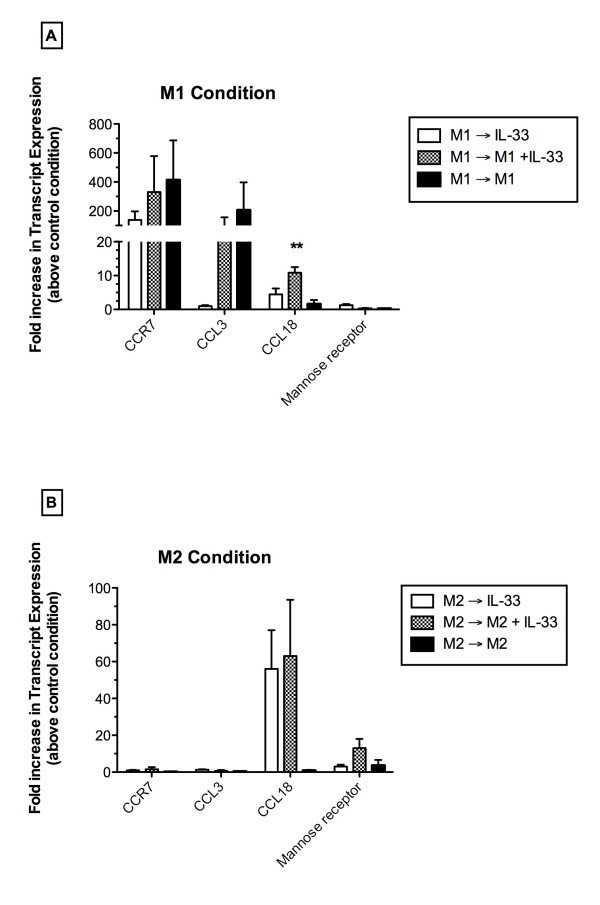
**Effect of IL-33 on human macrophages previously exposed to either M1 or M2 conditions**. M1- (i.e. CCR7 and CCL3) and M2- (i.e. CCL18 and mannose receptor) associated markers in human macrophages previously exposed to M1 (**A**) or M2 (**B**) conditions for 24 h, which were secondarily challenged with IL-33 alone (10 ng/ml), IL-33 + M1 or M2 polarizing mediators, or M1 or M2 polarizing mediators for an additional 24 h. Results are expressed as fold increase in transcript levels compared with levels of these transcripts in human macrophages exposed to media alone (i.e. control condition). Data shown are mean ± SEM, and these data are representative of three separate experiments with 3 donor macrophage lines. ** P ≤ 0.01 compared with transcript levels in appropriate control macrophages.

To investigate the effect of IL-33 on human macrophages following their exposure for 24 h to either M1 or M2 polarizing conditions, IL-33 alone, IL-33 with M1 or M2 polarizing mediators, or M1 or M2 polarizing mediators alone were added for an additional 24 h to primary human macrophage cultures. As shown in Figure 6A, the addition of IL-33 alone macrophages exposed to M1 mediators did not induce the expression of CCR7 to the same magnitude as that observed in M1 polarized cultures exposed to M1 mediators with IL-33 or M1 mediators. Similar results were observed with CCL3 transcript expression although IL-33 alone did not induce CCL3 transcript expression in M1 polarized human macrophages (Figure 6A). However, the presence of IL-33 with M1 polarizing mediators significantly increased the expression of the M2 marker CCL18 in macrophages compared with M1 macrophages alone (Figure 6A). In cultures of M2 polarized macrophages, transcript levels of CCL18 were markedly induced after the secondary addition of IL-33 alone or IL-33 with M2 mediators for an additional 24 h (Figure 6B). Interestingly, the secondary addition of M2 mediators did not further enhance CCL18 expression in M2 polarized macrophages (Figure 6B). The effect of IL-33 on mannose receptor transcript expression was also examined and similar results were observed; the secondary addition of the combination of IL-33 and M2 mediators further enhanced mannose receptor expression on M2 polarized macrophages. Overall, these data suggest that IL-33 appears to promote M2 chemokine expression in polarized macrophages since it promoted CCL18 in M1 human macrophages and appears to further enhance the expression of M2 markers in M2-polarized macrophages.

## Discussion

The present study demonstrates that human macrophages express ST2 and respond to the presence of IL-33. The expression of ST2, particularly the transmembrane form of this receptor, and to a lesser extent the soluble version of this receptor, was affected by the polarization of human macrophages either toward the expression of either M1 or M2 chemokine markers. Macrophages also showed differential responses to IL-33, which appeared to depend on the activation status of these cells. Naïve macrophages responded to IL-33 through the elaboration of M1 chemokine markers. Conversely, when added to M1 or M2 polarizing or polarized conditions, this IL-1 like cytokine promoted the expression of M2 chemokine markers in human macrophages. Together these findings suggest that there is a dynamic range of human macrophage responses to IL-33.

The ST2 gene encodes for two distinct isoforms: ST2L and sST2. ST2L is the transmembrane form of ST2 and is the active IL-33-signaling receptor while the soluble form of this receptor is thought to function as a soluble decoy for IL-33. Both ST2 isoforms are expressed by a number of innate and adaptive immune cells and their expression is altered by cytokine signals (including IL-33 itself) in both types of immune cells [17]. Regarding mouse macrophages, both forms of ST2 have been described in these cells although we have previously observed that ST2L levels are higher in alternatively activated or M2 mouse macrophages [24]. These data are consistent with the observation that exogenous IL-33 amplifies the polarization of alternatively activated macrophages [25]. In the present study, both ST2L and sST2 transcript and protein expression were examined in human macrophages. Both forms of ST2 were present in naïve human macrophages although sST2 was never detected in the cell-free supernatants from these cultures (data not shown). Both M1 and M2 polarizing mediators promoted the transcript expression of ST2L but not sST2 whereas the M2 polarizing conditions increased protein expression for both forms of ST2. These data are consistent with previous mouse macrophage data and highlight that ST2 expression is prominent on human M2 macrophages, but it is also expressed by naïve and M1 macrophages as well.

In contrast to earlier findings that functional IL-33 was a product of caspase-1 cleavage [9], it is now established that this cytokine is active without proteolysis and is inactivated by apoptotic caspases [8-10,26]. Full length IL-33 (IL-33^1-270^) is released from necrotic cells [8] or by activated mouse macrophages [27] independent of caspase or calpain activity. A truncated version of IL-33 (IL-33^112-270^) is formed via the activity of caspase 1 but it appears to lack the full potency of full length IL-33 [10]. At the time these studies were undertaken, recombinant commercial sources of IL-33 were limited to IL-33^112-270 ^thus this was the form of IL-33 used in cultures of human macrophages. Key observations from the present study included the M1 skewing effect of recombinant IL-33 on naïve human macrophages as evidenced by enhanced CCL3 expression. The inhibitory effect of IL-33 on LPS-induced CCL18 expression was consistent with the documented inhibitory effect of ST2 activation on TLR4 signaling [23,28]. However, IL-33 had no inhibitory effect on the expression of CCL3 by naïve human macrophages, data that was consistent with studies in mouse macrophages indicating that IL-33 did not inhibit LPS activation of these cells [18]. Thus, the addition of recombinant IL-33 to naïve human macrophages appeared to skew these cells toward the expression of M1 chemokine markers.

When added to macrophage cultures containing polarizing or polarized conditions, it was apparent that IL-33 had a divergent effect from that observed in cultures of naïve human macrophages. Specifically, IL-33 promoted or amplified M2-associated markers such as CCL18 and mannose receptors in these cells. These data are consistent with a previously published study showing that IL-33 amplifies the polarization of alternatively activated or M2 mouse macrophages [25] and contributes to Th2 responses [14,29]. At present, an explanation of the M2 skewing properties of IL-33 in polarizing or polarized human macrophages is not clear, but further studies will be directed at exploring the role of CCL18 in this process. CCL18 has no known mouse homolog and it has been described as a potent inducer of alternative activation in human macrophages thereby promoting fibrotic responses in the lung [30,31]. Thus, IL-33 uniquely promoted the expression of M2 chemokine markers in macrophages previously exposed to M1 or M2 conditions.

## Conclusions

this study addressed the effect of IL-33 on the activation and polarization of primary human macrophages. These findings point to a dual effect of IL-33 that depends on the activation status of these cells, findings that are consistent with those of Smithgall and colleagues [20] who demonstrated that IL-33 amplified both Th1 and Th2 type responses in human immune cells. Thus, the present findings highlight the complex immunomodulatory role of this cytokine on human macrophages.

## Authors' contributions

ADJ, SRA, and AJH carried out all the experiments described herein. WGF coordinated the collection of unused diagnostic bone marrow samples used herein. ADJ, SLK, KED, AD, and CMH participated in the design and coordination of the present study. ADJ and CMH wrote the manuscript. All authors read and approved the final manuscript.
